# Transcriptome analyses of reprogrammed feather / scale chimeric explants revealed co-expressed epithelial gene networks during organ specification

**DOI:** 10.1186/s12864-018-5184-x

**Published:** 2018-10-29

**Authors:** Yung-Chih Lai, Ya-Chen Liang, Ting-Xin Jiang, Randall B. Widelitz, Ping Wu, Cheng-Ming Chuong

**Affiliations:** 1 Integrative Stem Cell Center, China Medical University Hospital, China Medical University, Taichung, 40402 Taiwan; 2 Department of Pathology, Keck School of Medicine, University of Southern California, Los Angeles, CA 90033 USA; 3 Research Center for Developmental Biology and Regenerative Medicine, National Taiwan University, Taipei, 10617 Taiwan; 4 Center for the Integrative and Evolutionary Galliformes Genomics, National Chung-Hsing University, Taichung, 40227 Taiwan

**Keywords:** Feather, Scale, Ptilopody, Evo-devo, Regionalization, Recombination, RNA-Seq, ATAC-Seq

## Abstract

**Background:**

The molecular mechanism controlling regional specific skin appendage phenotypes is a fundamental question that remains unresolved. We recently identified feather and scale primordium associated genes and with functional studies, proposed five major modules are involved in scale-to-feather conversion and their integration is essential to form today’s feathers. Yet, how the molecular networks are wired and integrated at the genomic level is still unknown.

**Results:**

Here, we combine classical recombination experiments and systems biology technology to explore the molecular mechanism controlling cell fate specification. In the chimeric explant, dermal fate is more stable, while epidermal fate is reprogrammed to be similar to the original appendage type of the mesenchyme. We analyze transcriptome changes in both scale-to-feather and feather-to-scale transition in the epidermis. We found a highly interconnected regulatory gene network controlling skin appendage types. These gene networks are organized around two molecular hubs, β-catenin and retinoic acid (RA), which can bind to regulatory elements controlling downstream gene expression, leading to scale or feather fates. ATAC sequencing analyses revealed about 1000 altered widely distributed chromatin open sites. We find that perturbation of a key gene alters the expression of many other co-expressed genes in the same module.

**Conclusions:**

Our findings suggest that these feather / scale fate specification genes form an interconnected network and rewiring of the gene network can lead to changes of appendage phenotypes, acting similarly to endogenous reprogramming at the tissue level. This work shows that key hub molecules, β-catenin and retinoic acid, regulate scale / feather fate specification gene networks, opening up new possibilities to understand the switches controlling organ phenotypes in a two component (epithelial and mesenchyme) system.

**Electronic supplementary material:**

The online version of this article (10.1186/s12864-018-5184-x) contains supplementary material, which is available to authorized users.

## Background

Regional specificity is a long-standing, yet unresolved, biological question. Skin appendages provide an excellent system to explore regional specific differentiation, because amniotes display a large spectrum of integumentary appendage types in different body regions [1–4]. The local modifications of skin appendages allow specification into different forms, including hair, feathers, scales, claws, teeth and a range of glands. Specialized skin appendages play assorted functions (e.g. endothermy, protection and communication), allowing animals to adapt to diverse environments [5]. The chicken is the most intensively studied species to explore molecular principles of skin regionalization, because of the accessibility of chick embryos to experimentation and analysis and because of the marked distinction between the feathered dorsum and the scaled foot [6]. Classical experiments based on recombination of dermis and epidermis from distinct body regions revealed that dermis carries region-specific properties that determine appendage types [7, 8]. However, the molecules that are responsible for the dermal inductive capabilities and for the epidermal plastic response is scanty.

Scale to feather transition (i.e. ptilopody) has been found to be caused by a range of molecules that seem to involve different signaling pathways. For instance, over-expression of β-catenin [9] and Delta1 [10], treatment with retinoic acid [11, 12], repression of BMPR1B [13], and non-specific administration of bromodeoxyuridine (BrdU) [14], each can induce scale to feather conversions, although the extent of conversion can differ between these agents. Moreover, an ectopic feather-bearing skin can be induced by Noggin and Shh-expressing cells [15]. Feathers can be induced on scaleless mutant skin by FGF2 [16]. Until today, there is no simple model to explain why so many molecules can make feathered feet (ptilopody). More recently, we have found five novel molecules (Sox2, Zic1, Grem1, Spry2 and Sox18) expressed in the mesenchyme that have the ability to convert scale forming skin toward feather morphogenesis [17]. Each of these molecules seems to act along a different molecular pathway toward the morphological conversion of scales to feathers. Based on these findings, we postulate these molecular modules are part of a larger morpho-regulatory gene network that may have been co-opted in evolution to generate diverse appendage types, in this case, feathers and scales [17]. It should be noted avian scales include tarso-metatarsal scutate scale and plantar reticulate scales [2] and we only study scutate scales in this paper.

In this study, we further study changes in molecular expression that take place after epithelial-mesenchymal recombination of feather and scale tissues. Systemic molecular expression was profiled using RNA-Seq [18] to explore the genomic-wide molecular changes related to morphological transitions produced in the tissue recombination studies. We found dermis is more stable than the epidermis in the chimeric recombinants. Furthermore, we use ATAC-Seq [19] to examine sites of transcription factor binding at locations of open chromatin. The results show two hubs, beta-catenin signaling and retinoic acid signaling, to be in the epidermal gene network, responding to and controlling the regional-specific integumentary appendage organ phenotypes.

## Result

### Control of feather / scale phenotypes by mesenchyme is also demonstrated by transcriptome analyses

**Fig. 1:**
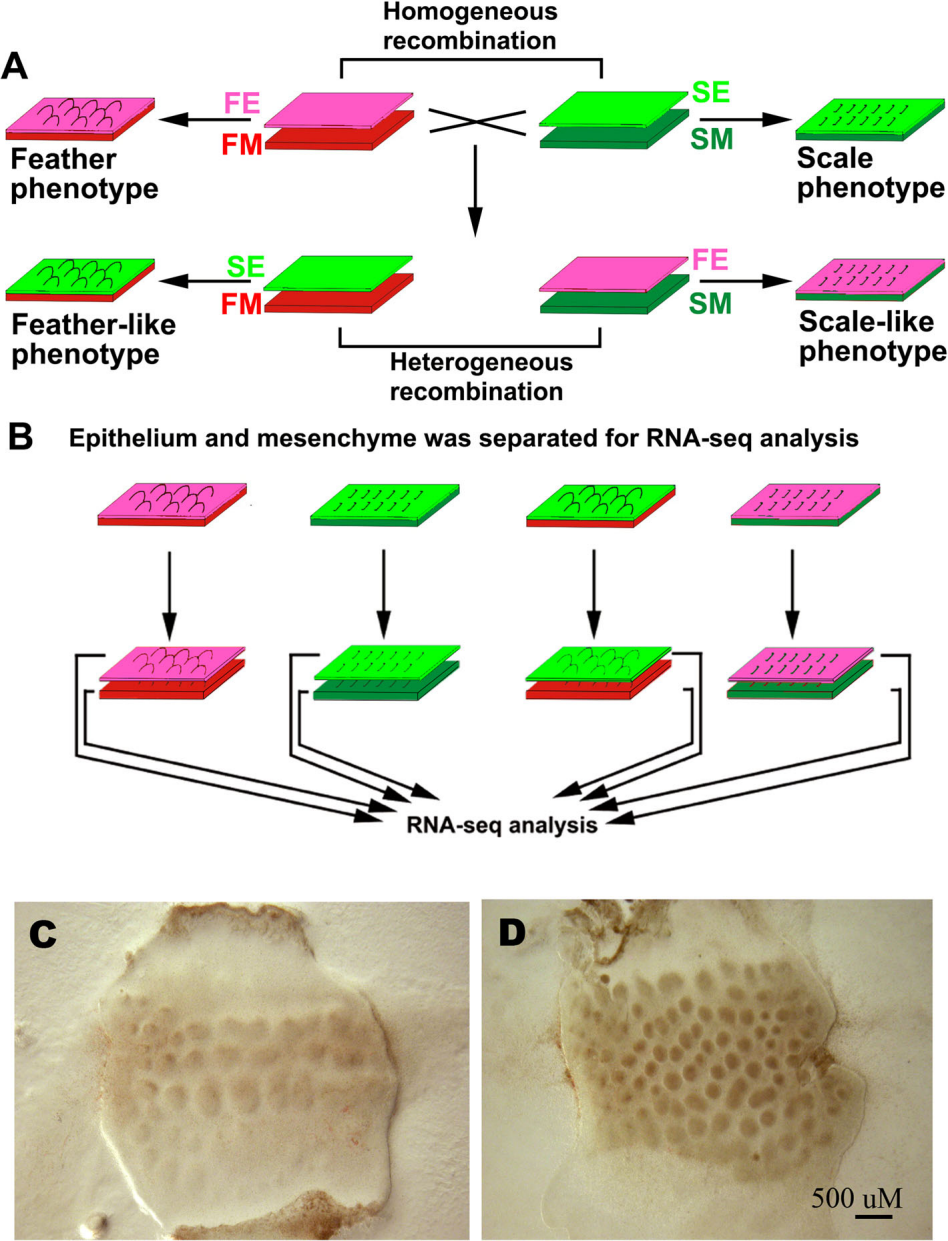
Experimental design for feather / scale recombination and RNA-Seq. **a**. Placode stage feather and scale forming skin were separated to epithelium and mesenchyme to perform recombination experiment. **b**. After 3 days in culture, epithelium and mesenchyme were separated again for RNA-Seq analysis. **c**. A scale-like phenotype from an E7 feather epithelium with E9 scale mesenchyme (FE/SM) culture. **d**. A feather-like phenotype from an E9 scale epithelium with E7 feather mesenchyme (SE/FM) culture. Both recombined skins were then cultured in an incubator at 37 °C, 5% CO_2_ and photographed under a Nikon C-DSD115 dissection microscope. The pictures were taken under live skin culture conditions without any staining. Because the lighting was from the beneath the bottom of the culture, the brown color was caused by the culture media and the lighting. FE, feather epithelium; FM, feather mesenchyme; SE, scale epithelium; SM, scale mesenchyme

In chicken feathers and scales, region-specific appendage identities are determined by epithelial-mesenchymal interactions [7, 8]. To determine the effects of epithelium and mesenchyme on gene expression, we conducted E7 feather and E9 scale recombination. Both E7 feathers and E9 scales are at the placode stage of development. E7 feather skin was separated to epithelium (FE) and mesenchyme (FM). Similarly, E9 scale skin was separated to epithelium (SE) and mesenchyme (SM). Homotypic-recombination of FE/FM (E7 feather epithelium with E7 feather mesenchyme) or SE/SM (E9 scale epithelium with E9 scale mesenchyme) are used as controls, whereas heterogeneous-combinations FE/SM (E7 feather epithelium with E9 scale mesenchyme) or SE/FM (E9 scale epithelium with E7 feather mesenchyme) are used as experimental groups (Fig. 1b). We independently collected epithelium and mesenchyme from all samples with biological replicates after 3 days in culture for gene profiling analysis (Additional file 1: Table S1). In homotypic-recombination experiments, feathers developed when both epithelium and mesenchyme originate from feather forming regions (Fig. 1a), and scales developed when both epithelium and mesenchyme originate from scale forming regions (Fig. 1a). In the heterogeneous-recombination experiment, when E9 scale epithelium was recombined with E7 feather mesenchyme, feather-like appendages developed after 3 days of culture (Fig. 1a and d). Conversely, scale-like appendages developed when E7 feather epithelium was recombined with E9 scale mesenchyme after 3 days of culture (Fig. 1a and c). These recombination experiments are consistent with previous recombination experiments, which suggest that mesenchyme, not epithelium, determines skin appendage fates.

**Fig. 2:**
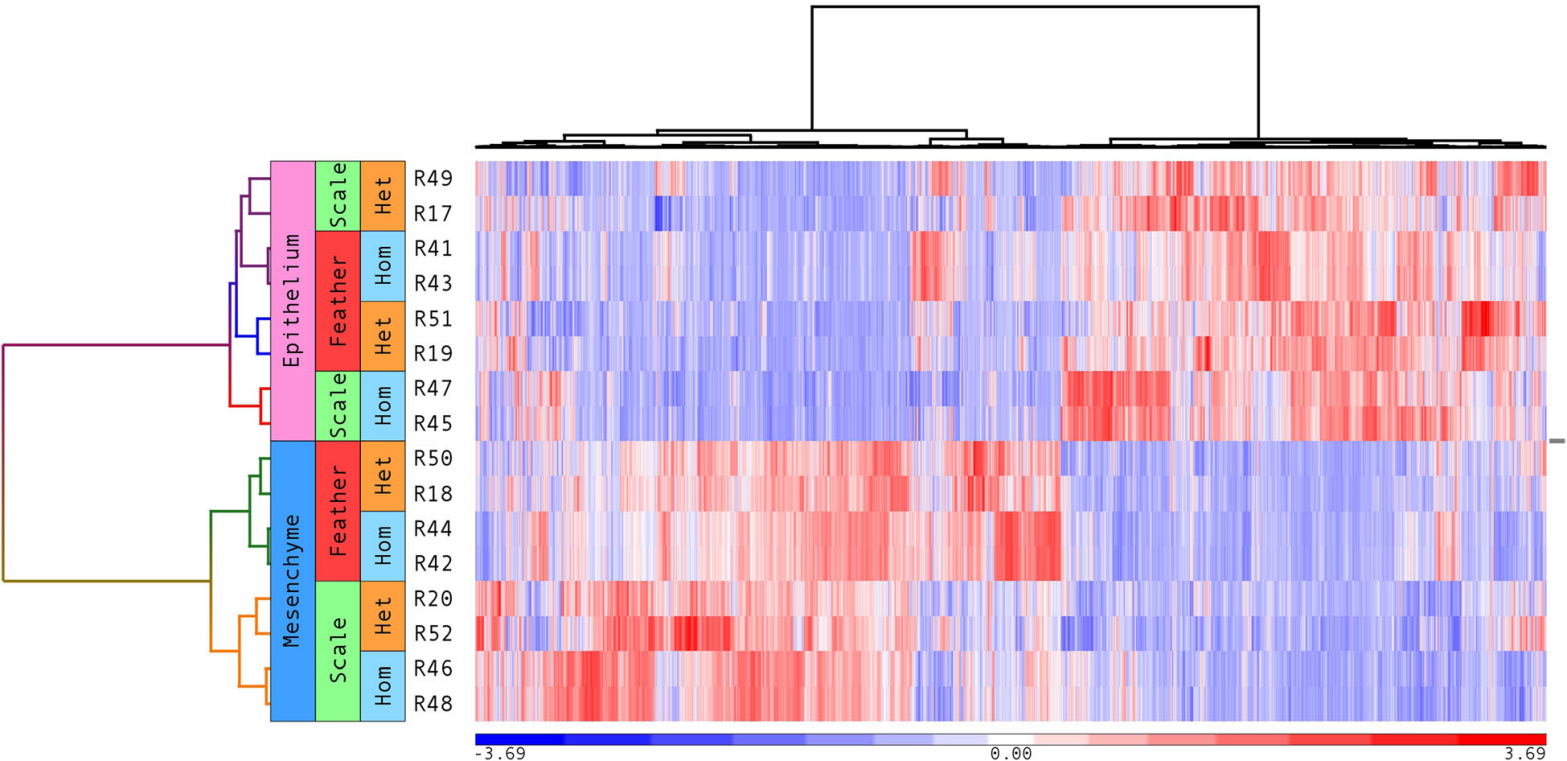
Hierarchical cluster analysis of transcription profiles on recombined and control samples. This analysis is based on 12,899 genes. Heterotypic recombined epithelial scale samples are clustered with homotypic recombined epithelial feather samples. In contrast, heterotypic recombined mesenchymal scale samples are clustered with homotypic recombined mesenchymal scale samples. The epithelium shows plasticity in responding to mesenchymal relatively stable signals

Non-biased hierarchical clustering of RNA-Seq samples demonstrates that epithelium and mesenchyme changes are grouped into two distinct clusters (Fig. 2). Recombined samples containing mesenchyme of feather origin evaluated for both FE/FM (feather phenotype) and SE/FM (feather-like phenotype) grouped together. The same trend was found in recombined samples containing mesenchyme originating from scales in SE/SM (scale phenotype) and FE/SM (scale-like phenotype). In contrast, results from epithelium samples did not follow a similar trend as seen for the mesenchyme. After FE/SM or SE/FM recombination, expression profiles of the heterotypic recombined tissues did not group with their homotypic recombination controls. For example, the SE/FM did not group with SE/SM gene expression profile, but instead grouped with FE/FM profile. This finding implies that the gene profile of the original scale epithelium combined with feather mesenchyme has been shifted towards a feather profile. The gene profile shift is more dramatic in epithelium than in mesenchyme, which indicates that epithelial phenotypes were determined by mesenchymal signaling.

### Identification of epithelium scale-to-feather transition genes (SFT) and feather-to-scale transition genes (FST)

**Fig. 3:**
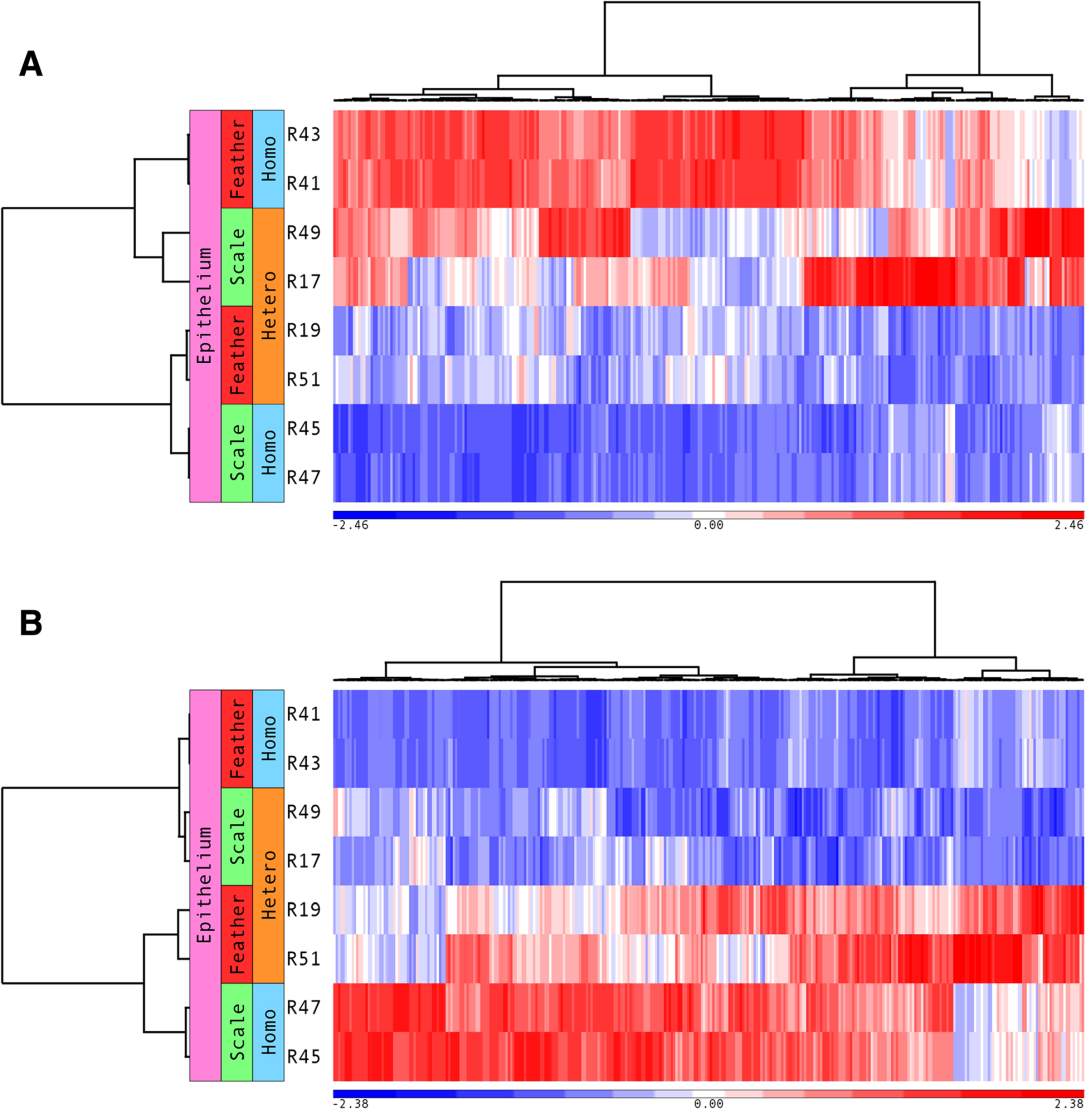
Hierarchical cluster analysis of genes involved in scale-to-feather and feather-to-scale transition. **a**. The co-transcription of 303 epithelium scale-to-feather transition (SFT) genes. SFT genes are up-regulated in homotypic recombined feathers and heterotypic recombined scale epithelium samples. **b**. The co-transcription of 327 epithelium feather-to-scale transition (FST) genes. FST genes are up-regulated in homotypic recombined scale and heterotypic recombined feather epithelium samples

We focused on gene expression changes that occur in the epithelium after recombination to identify genes that may be involved in producing either feathers or scales. First, we identified 1,261 differentially expressed genes in the epithelium between SE/FM (feather-like phenotype) and SE/SM (scale phenotype) recombined samples, including 668 up-regulated and 593 down-regulated genes in SE/FM compared to SE/SM samples; 3,282 differentially expressed genes in the epithelium between FE/SM (scale-like phenotype) and FE/FM (feather phenotype) samples, including 1,805 up-regulated and 1,477 down-regulated genes in FE/SM compared to FE/FM samples. Furthermore, using the above differentially expressed genes, we identified 303 feather specific genes (Additional file 2: Table S2). These genes are up-regulated in the epithelium of both FE/FM and SE/FM recombinations. At the same time, they are down-regulated in the epithelium of SE/SM and FE/SM recombinations (Fig. 3a). These genes are highly expressed in feather and feather-like phenotypes and expressed at low levels in scale and scale-like phenotypes; hence we call them epithelium scale-to-feather transition (SFT) genes. By contrast, in the epithelium of SE/SM and FE/SM recombinations, we identified 327 epithelium feather-to-scale transition (FST) genes (Additional file 3: Table S3). These genes are up-regulated in scale and scale-like epithelium, and down-regulated in feather and feather-like epithelium (Fig. 3b). Whereas in mesenchyme, we only identified 106 scale-to-feather transition (SFT) genes, and 71 feather-to-scale transition (FST) genes based on the same criteria.

### The most highly interconnected nodes in the epidermal gene networks are β-catenin and retinoic acid (RA)

We utilized Ingenuity Pathway Analysis (IPA) to explore molecular networks identified in gene lists from the SFT and FST experiments. Both SFT and FST gene lists are enriched with skin-associated functions (Additional file 4: Table S4A and S4B), which suggests these genes may play an important role in skin appendage determination and morphogenesis.

**Fig. 4:**
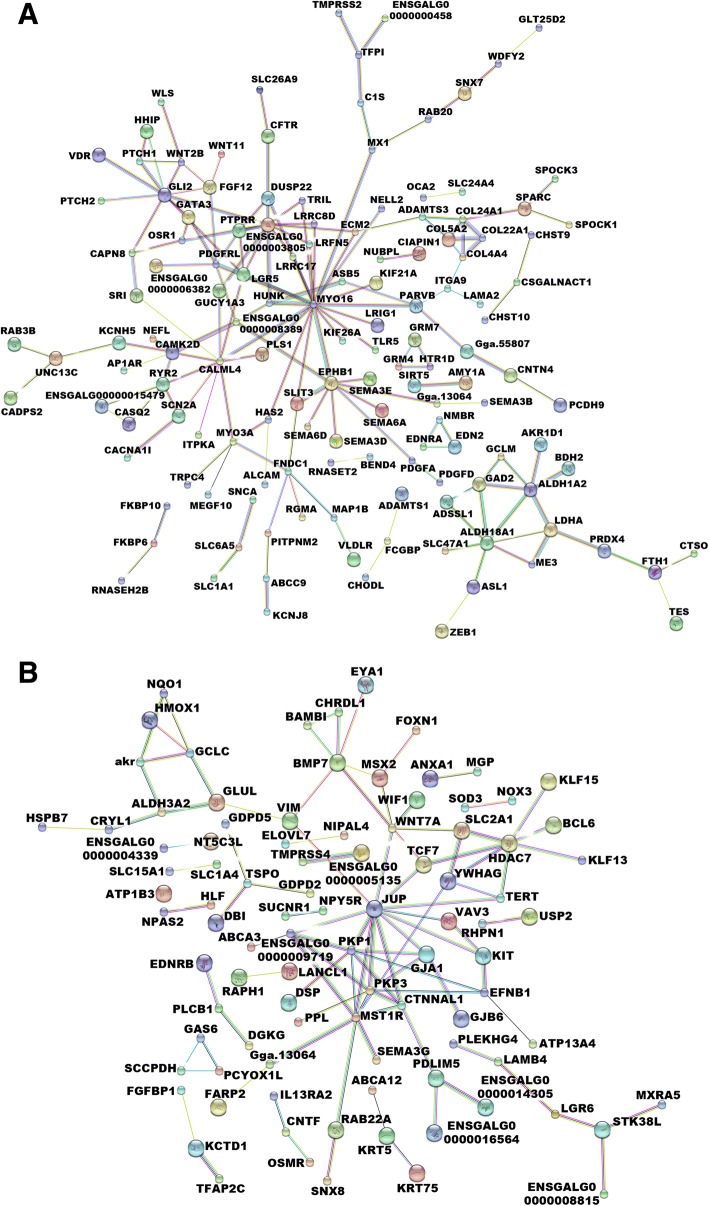
Both SFT and FST genes form tightly interconnected modules. **a** Epidermal scale-to-feather transition (SFT) genes form a tightly interconnected module (STRING, number of edges: 169, expected number of edges: 119, PPI enrichment *p*-value = 1.14 X 10^− 5^). **b** Epidermal feather-to-scale transition (FST) genes form a tightly interconnected module (STRING, number of edges: 101, expected number of edges: 62, PPI enrichment p-value = 3.57 X 10^− 6^). Edges are protein–protein interactions in the STRING database. Thicker lines indicate higher strength of data supporting these interactions. Unidentified and unlinked genes are not shown. The color used for nodes has no particular meaning

**Fig. 5:**
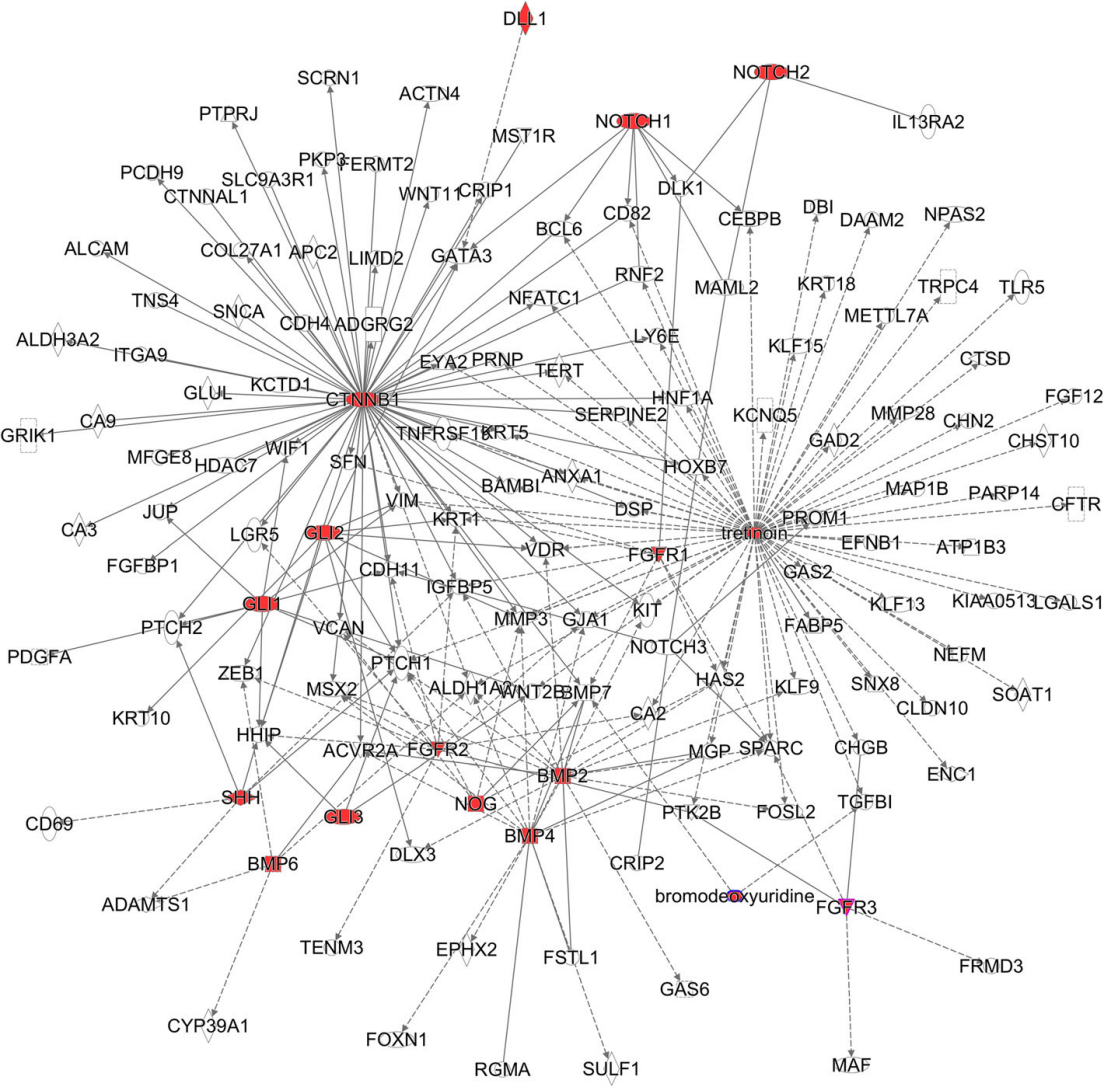
IPA Path Explorer identified a highly interconnected feather-scale co-expression network regulated by β-catenin and all-trans retinoic acid (RA, tretinoin) hubs. Red nodes are genes or chemicals involved in the formation of feathered feet (ptilopody) supported by the literature. White nodes are SFT and FST genes co-expression between feather and scale phenotypes based on recombination experiments. Every line represents a literature-supported relationship between a red and a white node. Solid and dashed lines indicate direct and indirect interactions, respectively. It shows that β-catenin and all-trans retinoic acid (RA, tretinoin) are two key hubs, because most of SFT and FST genes could be regulated by them

We further analyzed the gene network with STRING [20] and found that these genes can form interconnected networks. For epidermal scale-to-feather transition (SFT) genes, there are 119 expected interactions in the STRING database. However, we found there are 169 possible protein-protein interactions (Fig. 4a, PPI enrichment *p*-value = 1.14 X 10^− 5^). Similarly, we found 101 possible protein-protein interactions more than the 62 expected interactions for epidermal FST genes (Fig. 4b, PPI enrichment p-value = 3.57 X 10^− 6^). These results show that SFT and FST genes form tightly linked modules to determine skin appendage fate. Moreover, using IPA Path Explorer, we found that many SFT and FST genes are regulated by some key developmental molecules, involving Wnt/β-catenin (CTNNB1), all-trans retinoic acid (RA, Tretinoin), Sonic hedgehog (SHH), Notch, Bone Morphogenetic Protein (BMP), and Fibroblast Growth Factor (FGF) pathways (Fig. 5). Among them, β-catenin and all-trans retinoic acid (RA, Tretinoin) appear to be two key hubs. That is encouraging since β-catenin and retinoic acid can cause scale to feather conversion [9, 11]. This data suggests that these two molecules may be involved in regulating the expression of skin appendage associated genes (i.e. SFT and FST genes) leading to skin appendage specification and morphogenesis.

### β-Catenin and retinoic acid co-regulate regulatory elements involved in scale – feather reprogramming

Since β-catenin and retinoic acid (RA) are involved in scale to feather reprogramming conversion [9, 11] and these two signaling molecules are known to induce expression of a number of downstream genes [21, 22], we explored sites where they bind in open chromatin to regulate downstream gene expression. For this purpose, we performed ATAC-Seq of scale samples after ectopic β-catenin expression or all-trans retinoic acid (RA, Tretinoin) treatment (Additional file 5: Table S5). This method enables us to identify sites of open chromatin regions in response to the perturbation. E3 chicken embryos were transduced with RCAS-β-catenin [9]. E9 chicken embryos were treated with all-trans retinoic acid (RA, Tretinoin) [23]. RCAS-GFP treated samples was used as the control. Metatarsal skin was collected at E12 for ATAC-Seq analysis [19].

**Fig. 6:**
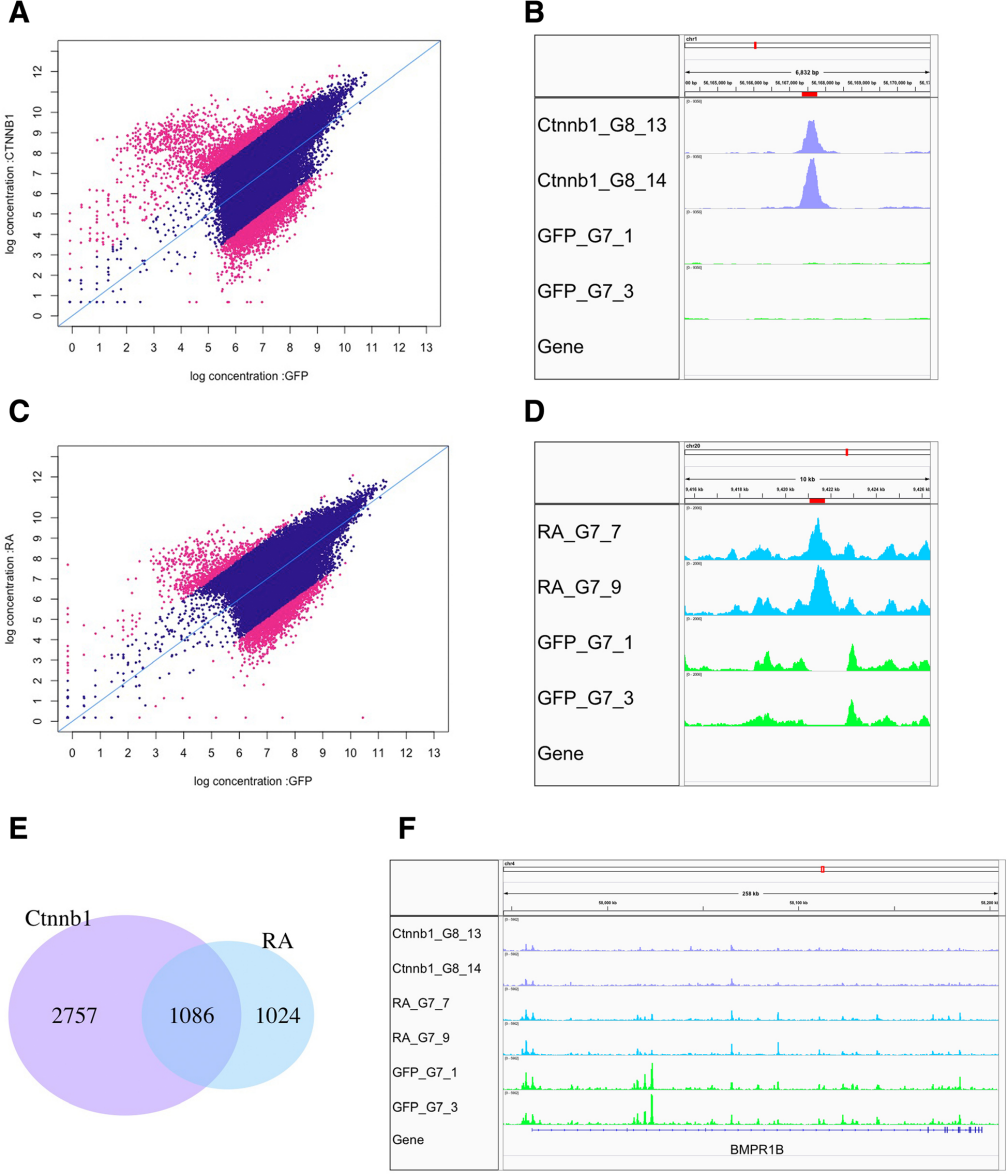
ATAC-Seq analysis reveals exemplary active and inhibited chromatin regions after β-catenin and RA perturbation. **a**. 3,844 significantly enriched ATAC-Seq peaks between RCAS-β-catenin and control. Magenta points are significantly differentially enriched peaks, and blue points are non-differentially enriched peaks. **b**. Example of ectopic β-catenin induced open chromatin region. **c**. 2,100 significantly enriched ATAC-Seq peaks after RA treatment. Magenta points are significantly differentially enriched peaks, and blue points are non-differentially enriched peaks. **d**. Example of RA induced open chromatin region. **e**. 1,086 differentially enriched peaks overlapped between β-catenin and RA treated samples. **f**. ATAC-Seq profiles of BMPR1B gene. Regulatory elements near BMPR1B are inhibited by RCAS-Ctnnb1 and retinoic acid (RA), compared to RCAS-GFP. The first and second tracks are RCAS-Ctnnb1, the third and fourth tracks are retinoic acid (RA), and the fifth and sixth tracks are RCAS-GFP

First, we compared open chromatin regions between RCAS-β-catenin overexpressing samples and controls. Ectopic β-catenin expression induced 3,844 significantly differentially enriched peaks, including 1,918 increased and 1,926 decreased locations (Fig. 6a). One example of increased β-catenin binding site is shown in Fig. 6b (red bar, chr1: 56,167,340 bp - 56,167,776 bp). There are no annotated genes within the 100 kb window around the peak. We then also identified 2,110 significantly enriched peaks influenced by RA, including 727 increased and 1,383 decreased open chromatin regions (Fig. 6c). One example of increased open chromatin sites after RA treatment is shown in Fig. 6d (red bar, chr20: 9,421,061 bp - 9,421,753 bp). As noted above for the β-catenin binding site, there are no annotated genes within the 100 kb window around this peak. Interestingly, we found 1,086 regulatory elements could be co-regulated by both β-catenin and RA (Fig. 6e). 51% of retinoic acid (RA) perturbed regulatory elements can be influenced by β-catenin. Similarly, 28% of β-catenin perturbed regulatory elements can be influenced by retinoic acid (RA). The result suggests the binding sites for β-catenin and retinoic acid response elements (RARE) could be in close proximity to enhancers, promoters, insulators, and so on. For instance, regulatory elements near and within BMPR1B are inhibited by both RCAS-β-catenin and retinoic acid (RA) (Fig. 6f). This finding that many feather-scale associated regulatory elements were co-regulated by β-catenin and RA may explain why similar phenotypes (i.e. ptilopody) are obtained by both treatments.

## Discussion

The feather is a novel integumentary organ that appeared during the evolution of dinosaurs – birds [2, 5, 24, 25]. Dhouailly has proposed a hypothesis stating that scales appeared secondarily from feathers during avian evolution [26]. She suggests that the default developmental program of avian epithelium will produce feather follicles. In this model, the foot mesenchyme suppresses feather development potential, and induces scale formation at the same time. Yet, to date, molecular agents have only been able to convert scales to feathers not feathers to scales. For example, RA treatment [11, 12], Wnt/β-catenin signaling activation [9], Notch/Delta pathway activation [10], or BMP pathway suppression [13] can convert scales to feathers. Recently Sox 2, Sox 18, Zic1 are also shown to convert scale – feather fate to different degrees [17]. These works suggested a large epidermal gene network can respond to these molecular modulators and then control epidermal organ specification. In comparison to the rapid progress in the epigenetic process of reprogramming cell fate at a single cell level [27], much less is known about the re-wiring of gene networks during reprogramming of the multi-component organ fate specification, a much complex situation.

Here we explored the molecular basis for epithelial – mesenchymal interactions using a tissue recombination study. Our results show that a feather-like phenotype was produced when feather or scutate scale epithelium was placed in contact with feather mesenchyme, and a scale-like phenotype was produced from feather or scutate scale epithelium in contact with scale mesenchyme. Similarly, originally transparent corneal epithelial cells could respond to signals originating from hair-bearing mesenchyme to form cutaneous appendages, including hair follicles and sweat glands [28]. In addition, no extant birds have teeth. However, tooth-like structures in chick skin explants can be induced when mandibular epithelium was recombined with dorsal mesenchyme [29]. These findings suggest that the mesenchyme has the capacity to induce overlying epithelium to form diverse types of ectodermal organs. To ascertain which expressed molecules might underlie regional specificity between feathers and scales using RNA-Seq we identified gene networks within the epithelium that regulate the formation of feather and scale skin appendages. Because scale-to-feather transition (SFT) and feather-to-scale transition (FST) genes are enriched with skin and skin appendage functions (Additional file 4: Table S4), the coordinated expression of these genes could specify skin appendage fate determination. Consequently, the scale-formation network could be perturbed by many converters because each key genes in this network could impact the whole regulatory network. In our previous paper, we studied regional specificity by focusing on differential gene expression in the mesenchyme [17]. For that study, we used intact embryos and performed RNA-Seq analysis on isolated mesenchyme from feather forming and scutate scale forming regions. That study identified 5 previously unknown molecules (Sox2, Zic1, Grem1, Spry2 and Sox18). Misexpressing these molecules in early embryogenesis caused scale to feather conversion to different extents. Ectopic *Sox2* expression converted the normal scale forming region to a feather forming field. Expression of a constitutively active form of *Zic1* caused the formation of an irregular surface pattern that suggested possible invaginations. *Grem1* caused the expression of ridges on the surface of scales. *Spry2* converted the scale forming field to a feather forming field, but the distribution of feathers followed the scale distribution pattern. *Sox18* caused feather filaments to form at the distal edge of scutate scales. Since these genes are expressed in the mesenchyme, they did not appear in our analysis of epithelium genes. In addition, both Tbx5 and Pitx1 genes associated with ptilopody [30] are not in our epithelial SFT and FST genes. It could be because that they are more upstream regulators for limb identities and are not the dermal specification genes for skin appendage types.

We know that perturbing either β-catenin or RA can disrupt skin appendage formations, hence it appears that these signals are acting at the epigenetic level. The ability of key gene perturbation to influence other co-expressed genes in the same network needs to be confirmed by 3C-based experiments [31, 32]. Using gene network analysis, we determined that β-catenin and RA were key molecular hubs in the epithelium that regulate skin appendage morphogenesis. Their regulation of downstream genes via binding to specific regulatory elements was explored using ATAC-Seq analysis. Although our ATAC-Seq samples are isolated from whole skin tissues, the major regulatory elements are affected by ectopic β-catenin expression and/or treatment with RA occurred in the epithelium, not the mesenchyme. We showed that β-catenin expression in both the epithelium and mesenchyme act to control epithelial appendage morphogenesis [9]. In contrast, RA acts exclusively on the epithelium, to form feathers on chicken foot scales [33]. In the skin, a dominant negative type 1 BMP receptor (dnBMPR1B) can block BMP signaling, which caused metatarsal scales to form feather filaments [13]. Therefore, the down-regulation of BMPR1B via an epigenetic mechanism possibly controlled by both ectopic β-catenin expression and by RA treatments (Fig. 6f) may explain why both RCAS-β-catenin and RA treatments can each induce feathered feet. This is especially true since we found similar downstream targets are regulated by these two key molecular hubs (Fig. 5). Our findings suggest that both β-catenin and RA can induce ptilopody by blocking BMP signaling.

The work here shows we now have a handle to begin dissecting the epidermal gene network that has evolved during reptile – avian evolution. The gene network has multiple interfaces to cross-talk with other signaling pathways. The gene network is also able to control other scale-feather converting signaling networks. Our results support the notion that perturbation of only one key gene can influence expression of the whole gene network to regulate skin appendage fate determination. Yet, we still need to learn where the multiple signaling modules interact in hierarchy or in parallel in development and evolution. In the future, the use of single-cell RNA-Seq [34] and individual-specific molecular network analysis [35] will improve the resolution of the gene network results identified in this study.

## Conclusions

We report gene expression profiles for differentially expressed genes on feather / scale recombination experiments. The changes in transcriptomes suggest epidermis is more plastic and dermis is more stable, consistent with the idea that dermis has a dominant role in skin appendage phenotypes. We also identify a highly interconnected co-expressed gene regulatory network when new feather or scale phenotypes are forming. In addition, chromatin accessible profiles suggest common regulatory elements regulated by β-catenin and retinoic acid (RA) hubs. Our findings imply that underlying molecular and epigenetic networks control regional specific skin appendage phenotypes and set down the platform for further investigation of these mechanisms.

## Methods

### Epithelium / mesenchyme recombination

The recombination experiments were performed as described in Hughes et al. [8]. Chicken eggs were incubated at 37 °C in an incubator with humidity control. Chicken dorsal skins (feather bud at placode stage) were collected at E7 (H&H stage 31). Similarly, metatarsal skins (scutate scale at placode stage) were dissected at E9 (H&H stage 35). Epithelium and mesenchyme were separated in 2X calcium and magnesium free medium (CMF) at 4 °C and they were recombined on cell culture inserts (Additional file 1: Table S1). For recombined skins, both epidermis and dermis isolated from prospective feathered region, or both epidermis and dermis isolated from the prospective scaled region belong to homogenous-recombination, i.e. control experiments. In contrast, epidermis and dermis isolated from different body regions belong to heterogeneous-recombination, i.e. chimera experiments. After 3 days of skin explant culture in an incubator with 5% CO_2_ at 37 °C, when new feather or scale phenotypes are forming, the recombined epidermal and dermal tissues were separated again for total RNA extraction using TRIzol reagent (Invitrogen).

### RNA-Seq

Illumina TruSeq RNA sample preparation v2 kit was used for library preparation. The 50 bp single-end RNA-Seq samples were generated using an Illumina HiSeq 2000 sequencer in University of Southern California (USC) Molecular Genomics Core. The sequencing depth is 30.3 ± 1.7 million reads (from 27.9 to 33.4 million) for each sample (Additional file 1: Table S1). The chicken galGal4 assembly including un-placed and un-localized scaffolds and Ensembl Release81 annotation were downloaded from the UCSC Genome Browser on 2016.2.6 [36]. Keratin (KRT) gene annotation [37] and epidermal differentiation complex (EDC) gene annotation [38] were added into the Ensembl annotation manually. Low quality of sequencing bases were trimmed based on the Phred quality score (> 20) from both of the 5′- and 3′-ends of reads. After trimming, reads are discarded, if they are shorter than 30 bp, or have one or more ambiguous bases. The alignment, quantification, normalization, and differential expression analysis were performed by STAR 2.4.1d [39] through Partek Flow (Partek Inc.), htseq-count 0.6.0 [40], TMM [41], and edgeR 3 [42], respectively. Genes with count-per-million (CPM) values above 1 in at least two samples were retained, and genes with no or low expression levels were discarded. The edgeR developed an exact test for differential expression appropriate for the negative binomially distributed counts. False discovery rate (FDR) < 0.05 was set as a threshold to identify differentially expressed genes. The Ward’s method hierarchical clustering using squared Euclidean distances was performed using Partek Genomics Suite (Partek Inc.). The protein-protein interaction network was tested using the STRING 10.5 database [20]. The null hypothesis is that the genes were selected at random. A small PPI enrichment *p*-value indicates that these genes are not random and that the observed number of edges is significant (http://version10.string-db.org/help/getting_started/). The pathway enrichment analysis using Fisher’s exact test was analyzed through the use of IPA (QIAGEN Inc., https://www.qiagenbioinformatics.com/products/ingenuity-pathway-analysis) [43]. IPA Path Explorer is used to build a regulatory network for feather-scale co-expression genes regulated by β-catenin and retinoic acid (RA). RNA-Seq raw data were accessible at NCBI GEO (accession number: GSE111099, a part of SuperSeries: GSE111101) https://www.ncbi.nlm.nih.gov/geo/query/acc.cgi?acc=GSE111099.

### ATAC-Seq

Gene and chemical perturbation experiments are carried out as described in Wu et al. [17]. Briefly, E3 (H&H stage 18) chicken samples were injected with RCAS-β-catenin. RCAS-GFP was used as control. Retinoic acid (RA) was delivered to E9 eggs according the method of Dhouailly et al. [11]. E12 (H&H stage 38) metatarsal skins were sampled for ATAC-Seq using the protocol described in Buenrostro et al. [19]. All ATAC-Seq libraries were sequenced on an Illumina NextSeq 500 sequencer in the University of Southern California (USC) Molecular Genomics Core with 40 bp paired-end reads (Additional file 5: Table S5). Reads were aligned onto the galGal4 reference genome using bowtie2 [44]. Greater than 19 million reads were obtained for each library and reads mapping to mitochondrial DNA were excluded. Coverage tracks (i.e. bigWig format) were produced by deepTools2 [45]. Peaks were called for each sample using MACS2 [46] with parameters: --format BAMPE --gsize 930000000 --keep-dup 1 --qvalue 0.00001. Differential peaks were identified using the DiffBind [47]. All ATAC-Seq datasets were deposited in GEO under accession number GSE111098, a part of SuperSeries: GSE111101 https://www.ncbi.nlm.nih.gov/geo/query/acc.cgi?acc=GSE111098.

## Additional files

Additional file 1: **Table S1.** List of RNA-Seq samples. (DOCX 84 kb)

Additional file 2: **Table S2.** The 303 epithelium scale-to-feather transition (SFT) genes. Column A and B are Ensembl gene ID and gene symbol, respectively. From column C to column F are normalized gene expression levels (CPM) for sample R19, R51, R41, and R43. Column G, H, and I are log2 fold change, *p*-value, and false discovery rate (FDR) of epithelial feather samples between heterogeneous and homogeneous experiments. From column J to column M are normalized gene expression levels (CPM) for sample R17, R49, R45, and R47. Column N, O, and P are log2 fold change, p-value, and false discovery rate (FDR) of epithelial scale samples between heterogeneous and homogeneous experiments. (XLSX 88 kb)

Additional file 3: **Table S3.** The 327 epithelium feather-to-scale transition (FST) genes. Column A and B are Ensembl gene ID and gene symbol, respectively. From column C to column F are normalized gene expression levels (CPM) for sample R19, R51, R41, and R43. Column G, H, and I are log2 fold change, p-value, and false discovery rate (FDR) of epithelial feather samples between heterogeneous and homogeneous experiments. From column J to column M are normalized gene expression levels (CPM) for sample R17, R49, R45, and R47. Column N, O, and P are log2 fold change, p-value, and false discovery rate (FDR) of epithelial scale samples between heterogeneous and homogeneous experiments. (XLSX 89 kb)

Additional file 4: **Table S4.** IPA enriched pathways based on 303 SFT (A) and 327 FST genes (B) (DOCX 118 kb)

Additional file 5: **Table S5.** List of ATAC-Seq samples. (DOCX 51 kb)

## Abbreviations

ATAC-Seq: Assay for transposase-accessible chromatin using sequencing
E: embryonic day
FE: feather epithelium
FM: feather mesenchyme
FST: feather-to-scale transition genes
RA: retinoic acid
RNA-Seq: RNA sequencing
SE: scale epithelium
SFT: scale-to-feather transition genes
SM: scale mesenchyme

## References

[1] Wu P, Hou LH, Plikus M, Hughes M, Scehnet J, Suksaweang S, Widelitz RB, Jiang TX, Chuong CM (2004). Evo-devo of amniote integuments and appendages. Int J Dev Biol.

[2] Dhouailly D, Godefroit P, Martin T, Nonchev S, Caraguel F, Oftedal O. Getting to the root of scales, feather and hair: as deep as odontodes? Exp Dermatol. 2017:1–6. 10.1111/exd.13391 28603898

[3] Johansson JA, Headon DJ (2014). Regionalisation of the skin. Semin Cell Dev Biol.

[4] Li A, Lai YC, Figueroa S, Yang T, Widelitz RB, Kobielak K, Nie Q, Chuong CM (2015). Deciphering principles of morphogenesis from temporal and spatial patterns on the integument. Dev Dyn.

[5] Chuong CM, Wu P, Zhang FC, Xu X, Yu M, Widelitz RB, Jiang TX, Hou LH (2003). Adaptation to the sky: defining the feather with integument fossils from Mesozoic China and experimental evidence from molecular laboratories. J Exp Zool Part B.

[6] Widelitz RB, Jiang TX, Yu MK, Shen T, Shen JY, Wu P, Yu ZC, Chuong CM (2003). Molecular biology of feather morphogenesis: a testable model for evo-devo research. J Exp Zool Part B.

[7] Rawles ME (1963). Tissue interactions in scale and feather development as studied in dermal-epidermal recombinations. J Embryol Exp Morphol.

[8] Hughes MW, Wu P, Jiang TX, Lin SJ, Dong CY, Li A, Hsieh FJ, Widelitz RB, Chuong CM (2011). In search of the Golden fleece: unraveling principles of morphogenesis by studying the integrative biology of skin appendages. Integr Biol.

[9] Widelitz RB, Jiang TX, Lu JF, Chuong CM (2000). Beta-catenin in epithelial morphogenesis: conversion of part of avian foot scales into feather buds with a mutated Beta-catenin. Dev Biol.

[10] Crowe R, Niswander L (1998). Disruption of scale development by Delta-1 misexpression. Dev Biol.

[11] Dhouailly D, Hardy MH, Sengel P (1980). Formation of feathers on chick foot scales - a stage-dependent morphogenetic response to retinoic acid. J Embryol Exp Morphol.

[12] Dhouailly D, Hardy MH (1978). Retinoic acid causes development of feathers in scale-forming integument of chick-embryo. Wilhelm Roux Arch Dev Biol.

[13] Zou HY, Niswander L (1996). Requirement for BMP signaling in interdigital apoptosis and scale formation. Science.

[14] Tanaka S, Sugiharayamamoto H, Kato Y (1987). Epigenesis in developing avian scales III. Stage-specific alterations of the developmental program caused by 5-bromodeoxyuridine. Dev Biol.

[15] Fliniaux I, Viallet JP, Dhouailly D (2004). Signaling dynamics of feather tract formation from the chick somatopleure. Development.

[16] Prin F, Dhouailly D (2004). How and when the regional competence of chick epidermis is established: feathers vs. scutate and reticulate scales, a problem en route to a solution. Int J Dev Biol.

[17] Wu P, Yan J, Lai YC, Ng CS, Li A, Jiang XY, Elsey RM, Widelitz R, Bajpai R, Li WH (2018). Multiple regulatory modules are required for scale-to-feather conversion. Mol Biol Evol.

[18] Mortazavi A, Williams BA, McCue K, Schaeffer L, Wold B (2008). Mapping and quantifying mammalian transcriptomes by RNA-Seq. Nat Methods.

[19] Buenrostro JD, Giresi PG, Zaba LC, Chang HY, Greenleaf WJ (2013). Transposition of native chromatin for fast and sensitive epigenomic profiling of open chromatin, DNA-binding proteins and nucleosome position. Nat Methods.

[20] Szklarczyk D, Franceschini A, Wyder S, Forslund K, Heller D, Huerta-Cepas J, Simonovic M, Roth A, Santos A, Tsafou KP (2015). STRING v10: protein-protein interaction networks, integrated over the tree of life. Nucleic Acids Res.

[21] Balmer JE, Blomhoff R (2002). Gene expression regulation by retinoic acid. J Lipid Res.

[22] Schuijers J, Mokry M, Hatzis P, Cuppen E, Clevers H (2014). Wnt-induced transcriptional activation is exclusively mediated by TCF/LEF. EMBO J.

[23] Chuong CM, Ting SA, Widelitz RB, Lee YS (1992). Mechanism of skin morphogenesis. II. Retinoic acid modulates axis orientation and phenotypes of skin appendages. Development.

[24] Xu X, Zhou Z, Dudley R, Mackem S, Chuong CM, Erickson GM, Varricchio DJ (2014). An integrative approach to understanding bird origins. Science.

[25] Chen CF, Foley J, Tang PC, Li A, Jiang TX, Wu P, Widelitz RB, Chuong CM, Lewin HA, Roberts RM (2015). Development, regeneration, and evolution of feathers. Annual review of animal biosciences. Vol. 3.

[26] Dhouailly D (2009). A new scenario for the evolutionary origin of hair, feather, and avian scales. J Anat.

[27] Wang YX, Bi Y, Gao SR (2017). Epigenetic regulation of somatic cell reprogramming. Curr Opin Genet Dev.

[28] Ferraris C, Chevalier G, Favier B, Jahoda CAB, Dhouailly D (2000). Adult corneal epithelium basal cells possess the capacity to activate epidermal, pilosebaceous and sweat gland genetic programs in response to embryonic dermal stimuli. Development.

[29] Chen YP, Zhang YD, Jiang TX, Barlow AJ, St Amand TR, Hu YP, Heaney S, Francis-West P, Chuong CM, Maas R (2000). Conservation of early odontogenic signaling pathways in Aves. Proc Natl Acad Sci U S A.

[30] Domyan ET, Kronenberg Z, Infante CR, Vickrey AI, Stringham SA, Bruders R, Guernsey MW, Park S, Payne J, Beckstead RB (2016). Molecular shifts in limb identity underlie development of feathered feet in two domestic avian species. elife.

[31] Kieffer-Kwon KR, Tang Z, Mathe E, Qian J, Sung MH, Li G, Resch W, Baek S, Pruett N, Grontved L (2013). Interactome maps of mouse gene regulatory domains reveal basic principles of transcriptional regulation. Cell.

[32] Li GL, Ruan XA, Auerbach RK, Sandhu KS, Zheng MZ, Wang P, Poh HM, Goh Y, Lim J, Zhang JY (2012). Extensive promoter-centered chromatin interactions provide a topological basis for transcription regulation. Cell.

[33] Cadi R, Dhouailly D, Sengel P (1983). Use of retinoic acid for the analysis of dermal epidermal interactions in the tarsometatarsal skin of the chick-embryo. Dev Biol.

[34] Zheng GXY, Terry JM, Belgrader P, Ryvkin P, Bent ZW, Wilson R, Ziraldo SB, Wheeler TD, McDermott GP, Zhu JJ (2017). Massively parallel digital transcriptional profiling of single cells. Nat Commun.

[35] Shimamura T, Imoto S, Shimada Y, Hosono Y, Niida A, Nagasaki M, Yamaguchi R, Takahashi T (2011). Miyano S. a novel network profiling analysis reveals system changes in epithelial-mesenchymal transition. PLoS One.

[36] Speir ML, Zweig AS, Rosenbloom KR, Raney BJ, Paten B, Nejad P, Lee BT, Learned K, Karolchik D, Hinrichs AS (2016). The UCSC genome browser database: 2016 update. Nucleic Acids Res.

[37] Ng CS, Wu P, Fan WL, Yan J, Chen CK, Lai YT, Wu SM, Mao CT, Chen JJ, Lu MYJ (2014). Genomic organization, transcriptomic analysis, and functional characterization of avian alpha- and beta-keratins in diverse feather forms. Genome Biol Evol.

[38] Strasser B, Mlitz V, Hermann M, Rice RH, Eigenheer RA, Alibardi L, Tschachler E, Eckhart L (2014). Evolutionary origin and diversification of epidermal barrier proteins in amniotes. Mol Biol Evol.

[39] Dobin A, Davis CA, Schlesinger F, Drenkow J, Zaleski C, Jha S, Batut P, Chaisson M, Gingeras TR (2013). STAR: ultrafast universal RNA-seq aligner. Bioinformatics.

[40] Anders S, Pyl PT, Huber W (2015). HTSeq-a Python framework to work with high-throughput sequencing data. Bioinformatics.

[41] Robinson MD, Oshlack A (2010). A scaling normalization method for differential expression analysis of RNA-seq data. Genome Biol.

[42] Robinson MD, Smyth GK (2008). Small-sample estimation of negative binomial dispersion, with applications to SAGE data. Biostatistics.

[43] Kramer A, Green J, Pollard J, Tugendreich S (2014). Causal analysis approaches in ingenuity pathway analysis. Bioinformatics.

[44] Langmead B, Salzberg SL (2012). Fast gapped-read alignment with bowtie 2. Nat Methods.

[45] Ramirez F, Dundar F, Diehl S, Gruning BA, Manke T (2014). deepTools: a flexible platform for exploring deep-sequencing data. Nucleic Acids Res.

[46] Zhang Y, Liu T, Meyer CA, Eeckhoute J, Johnson DS, Bernstein BE, Nussbaum C, Myers RM, Brown M, Li W (2008). Model-based analysis of ChIP-Seq (MACS). Genome Biol.

[47] Ross-Innes CS, Stark R, Teschendorff AE, Holmes KA, Ali HR, Dunning MJ, Brown GD, Gojis O, Ellis IO, Green AR (2012). Differential oestrogen receptor binding is associated with clinical outcome in breast cancer. Nature.

