# Supplementary material for: Transcriptome analyses of reprogrammed feather / scale chimeric explants revealed co-expressed epithelial gene networks during organ specification
Source: BMC Genomics. 2018 Oct 29;19:780. doi: 10.1186/s12864-018-5184-x (PMC6206740; doi:10.1186/s12864-018-5184-x)
Supplement: Supplementary file 1 — Table S1. List of RNA-Seq samples. (DOCX 84 kb) [file 12864_2018_5184_MOESM1_ESM.docx]

**Table S1. List of RNA-Seq samples**

| Name | Recombination | Sample source | Phenotype | Total sequencing reads |
| --- | --- | --- | --- | --- |
| R41 | FE/FM-replicate1 | E7-FE | Feather | 29,177,778 |
| R43 | FE/FM-replicate2 | E7-FE | Feather | 29,641,639 |
| R42 | FE/FM-replicate1 | E7-FM | Feather | 27,871,105 |
| R44 | FE/FM-replicate2 | E7-FM | Feather | 29,960,367 |
| R45 | SE/SM-replicate1 | E9-SE | Scale | 29,065,630 |
| R47 | SE/SM-replicate2 | E9-SE | Scale | 32,769,439 |
| R46 | SE/SM-replicate1 | E9-SM | Scale | 32,961,493 |
| R48 | SE/SM-replicate2 | E9-SM | Scale | 31,353,552 |
| R19 | FE/SM-replicate1 | E7-FE | Scale-like | 31,098,958 |
| R51 | FE/SM-replicate2 | E7-FE | Scale-like | 28,127,004 |
| R18 | FE/SM-replicate1 | E9-SM | Scale-like | 31,396,337 |
| R50 | FE/SM-replicate2 | E9-SM | Scale-like | 28,425,805 |
| R17 | SE/FM-replicate1 | E9-SE | Feather-like | 30,751,533 |
| R49 | SE/FM-replicate2 | E9-SE | Feather-like | 33,365,680 |
| R20 | SE/FM-replicate1 | E7-FM | Feather-like | 29,670,037 |
| R52 | SE/FM-replicate2 | E7-FM | Feather-like | 29,931,389 |

FE, E7 feather epithelium; FM, E7 feather mesenchyme;

SE, E9 scale epithelium; SM, E9 scale mesenchyme.
