# Supplementary material for: Transcriptome analyses of reprogrammed feather / scale chimeric explants revealed co-expressed epithelial gene networks during organ specification
Source: BMC Genomics. 2018 Oct 29;19:780. doi: 10.1186/s12864-018-5184-x (PMC6206740; doi:10.1186/s12864-018-5184-x)
Supplement: Supplementary file 4 — Table S4. IPA enriched pathways based on 303 SFT (A) and 327 FST genes (B) (DOCX 118 kb) [file 12864_2018_5184_MOESM4_ESM.docx]

**Table S4. IPA enriched pathways based on 303 SFT (A) and 327 FST genes (B).**

(A)

| Annotation | Gene |
| --- | --- |
| Formation of skin (P = 4.9 X 10^-3^) | CDH11, COL5A2, EDN2, GLI2, KRT18, LGR5, LRIG1, MAP3K12, PDGFA, PTCH1, PTCH2, VCAN, VDR |
| Formation of epidermis (P = 4.9 X 10^-3^) | CDH11, GLI2, LGR5, PDGFA, PTCH1, PTCH2, VCAN, VDR |

(B)

| Annotation | Gene |
| --- | --- |
| Formation of skin (P = 2.8 X 10^-10^) | ABCA12, AGO2, ALDH3A2, ANXA1, BMP7, CD109, DBI, DLX3, DSP, ELOVL4, EXPH5, FABP5, FOSL2, FOXN1, GJA1, IGFBP5, JUP, KIT, KRT5, MSX2, POU2F3, SFN, SPINK5, TFAP2C, TNFRSF19 |
| Morphology of stratum corneum (P = 6.4 X 10^-7^) | ELOVL4, GJA1, JUP, KRT1, PKP1, SLC9A1, SPINK5 |
| Formation of epidermis (P = 1.6 X 10^-6^) | ALDH3A2, CD109, DBI, DSP, ELOVL4, FABP5, FOXN1, GJA1, IGFBP5, KRT5, POU2F3, TFAP2C, TNFRSF19 |
| Morphology of hair (P = 4.9 X 10^-6^) | BMP7, DBI, EFNB1, FOXN1, JUP, MSX2, PKP1, PKP3, SOAT1, SPINK5 |
| Differentiation of skin (P = 8.4 X 10^-6^) | ABCA12, ANXA1, CD109, DLX3, DSP, FABP5, FOXN1, KIT, MSX2, POU2F3, SFN, SPINK5 |
| Differentiation of epidermal cells  (P = 1.7 X 10^-5^) | ABCA12, ANXA1, CD109, DLX3, DSP, FABP5, FOXN1, MSX2, POU2F3, SFN, SPINK5 |
| Growth of skin (P = 2.7 X 10^-5^) | ALDH3A2, CD109, JUP, KLF9, KRT10, MSX2, PKP1, PKP3, SFN, SLURP1, TERT, VAV3, VIM |
| Proliferation of epidermal cells  (P = 4.3 X 10^-5^) | ALDH3A2, CD109, JUP, KLF9, KRT10, PKP1, PKP3, SFN, SLURP1, TERT, VAV3 |
| Differentiation of keratinocytes (P = 4.6 X 10^-5^) | ABCA12, ANXA1, CD109, DLX3, DSP, FABP5, FOXN1, MSX2, POU2F3, SFN |
| Proliferation of keratinocytes  (P = 9.6 X 10^-5^) | ALDH3A2, CD109, JUP, KLF9, KRT10, PKP1, PKP3, SFN, SLURP1, VAV3 |
| Formation of hair follicle  (P = 2.6 X 10^-3^) | CD109, DBI, FOXN1, IGFBP5, TFAP2C, TNFRSF19 |
