# Supplementary material for: Transcriptome analyses of reprogrammed feather / scale chimeric explants revealed co-expressed epithelial gene networks during organ specification
Source: BMC Genomics. 2018 Oct 29;19:780. doi: 10.1186/s12864-018-5184-x (PMC6206740; doi:10.1186/s12864-018-5184-x)
Supplement: Supplementary file 5 — Table S5. List of ATAC-Seq samples. (DOCX 51 kb) [file 12864_2018_5184_MOESM5_ESM.docx]

**Table S5. List of ATAC-Seq samples**

| Name | Perturbation | Phenotype | Stage | Tissue | Total reads |
| --- | --- | --- | --- | --- | --- |
| G8_13 | RCAS-β-catenin | ptilopody | E12 | Metatarsal skin | 20,978,229 |
| G8_14 | RCAS-β-catenin | ptilopody | E12 | Metatarsal skin | 21,829,438 |
| G7_1 | RCAS-GFP | scale | E12 | Metatarsal skin | 19,715,578 |
| G7_3 | RCAS-GFP | scale | E12 | Metatarsal skin | 19,212,399 |
| G7_7 | Retinoic acid | ptilopody | E12 | Metatarsal skin | 22,994,792 |
| G7_9 | Retinoic acid | ptilopody | E12 | Metatarsal skin | 21,716,918 |
